# Tobacco smoking was positively associated with metabolic syndrome among patients with psoriasis in Shanghai: A cross-sectional study

**DOI:** 10.18332/tid/144228

**Published:** 2022-01-22

**Authors:** Lei Wei, Siting Chen, Yan Qiang, Le Kuai, Mi Zhou, Ying Luo, Yue Luo, Jiankun Song, Xiaoya Fei, Rui Zhang, Ning Yu, Wencheng Jiang, Xin Li, Ruiping Wang, Bin Li

**Affiliations:** 1 Department of Dermatology, Yueyang Hospital of Integrated Traditional Chinese and Western Medicine, Shanghai University of Traditional Chinese Medicine, Shanghai, China; 2 Clinical Research and Innovation Transformation Center, Shanghai Skin Diseases Hospital, Tongji University, Shanghai, China; 3 Institute of Dermatology, Shanghai Academy of Traditional Chinese Medicine, Shanghai, China; 4 Department of Dermatology, Shanghai Skin Diseases Hospital, Tongji University, Shanghai, China

**Keywords:** psoriasis, metabolic syndrome, tobacco smoking, association

## Abstract

**INTRODUCTION:**

A number of studies have reported a high correlation between psoriasis and metabolic syndrome (MetS), and tobacco smoking is one independent risk factor accounting for the increased prevalence both for psoriasis and MetS. However, few studies have been conducted to assess the effects of tobacco smoking on co-morbidities of psoriasis and MetS.

**METHODS:**

We conducted a cross-sectional study with 1014 psoriasis patients recruited from January to May 2021. Patients were recruited with a cluster survey method in Yueyang Hospital (affiliated with Shanghai University of Traditional Chinese Medicine) and Shanghai Skin Diseases Hospital (affiliated with Tongji University). Data were collected by face-to-face questionnaire interviews which included basic information, personal life habits, medical history, and clinical examinations. SPSS 24.0 was used for data analysis and a p<0.05 was considered statistically significant.

**RESULTS:**

The 1014 psoriasis patients were predominantly males (65.58%), with an average age of 45.98 years (IQR: 34.00–57.00). Of these, 25.74% (261) of psoriasis had MetS and 31.85% (323) were tobacco smokers. Male psoriasis patients had higher tobacco smoking prevalence than female patients. With increasing age and BMI, the prevalence of tobacco smoking among psoriasis patients increased dramatically (p<0.01). Logistic regression indicated that psoriasis patients with tobacco smoking had 1.78 times (95% CI: 1.21–2.60) the probability to have MetS than those without tobacco smoking, even adjusting for potential confounding factors. Moreover, smoking psoriasis patients with MetS consumed more cigarettes per day, with longer smoking duration, but with an older age of smoking initiation.

**CONCLUSIONS:**

The prevalence of tobacco smoking and MetS among psoriasis patients was high in Shanghai, and tobacco smoking was positively associated with the MetS among psoriasis patients. Clinicians should recommend psoriasis patients to abstain from tobacco smoking and provide tobacco cessation assistance regularly.

## INTRODUCTION

Psoriasis is an immune-mediated chronic inflammatory disease, and approximately 125 million people suffer from psoriasis worldwide^1^. Psoriasis vulgaris is the most common subtype of psoriasis, accounting for approximately 80–90% of all psoriasis cases^2^. Psoriasis vulgaris is characterized by the erythema and scaling on the skin. It also affects the patient’s joints, tissues, and multiple organs^3^. Thus, psoriasis vulgaris has been defined as a systemic disease that is co-morbid with multiple diseases such as metabolic, cardiovascular, or psychiatric disorders^4-6^. The co-morbidity of psoriasis and metabolic syndrome has attracted increasing attention from the clinician. Several studies have reported the co-prevalence of these two diseases, but the findings were inconsistent^7-9^.

Psoriasis is a hyper-proliferative inflammatory disease with a disorder of the immune systems. Multiple pathogenesis may cause recurrent morbidity in psoriasis, including genetic and environmental (e.g. tobacco smoking, microbial infections, drugs and stress) factors^10-12^. Metabolic syndromes (MetS) are a group of metabolic disorders involving systemic inflammation, insulin resistance, and obesity, with genetic and environmental underpinning also playing a critical role^13^. Several studies demonstrated that the common pathogenesis of psoriasis combined with metabolic syndrome may be some shared genetic links, environmental factors or inflammatory pathways^14^. For instance, a mechanistic study has reported that the psoriasis susceptibility (PSORS) loci PSORS^2-4^ is closely linked to susceptibility loci for metabolic syndrome and cardiovascular disease^15^. Another study indicated that IL-17A and its proinflammatory functions are also a significant cause of the comorbidity^16^.

Tobacco smoking is known as an inflammation-inducing behavior^17^. Smoking-induced oxidative stress and inflammation are treated as key mechanisms for smoking induced disease, which may cause a constant state of elevated inflammation levels in the body^18,19^. A number of studies have reported a high correlation between psoriasis and metabolic syndrome^20,21^, and smoking is one of the independent risk factors accounting for an increased prevalence both for psoriasis and metabolic syndrome^22,23^. However, few studies have been conducted to assess the effect of tobacco smoking on co-morbidities of psoriasis and metabolic syndrome. In this cross-sectional study, we aimed to understand the prevalence of tobacco smoking and metabolic syndrome among patients with psoriasis, and explore the association between tobacco smoking and metabolic syndrome among patients with psoriasis. Risk assessment of these potentially modifiable factors may be important for psoriasis management.

## METHODS

### Study design

We implemented a hospital-based cross-sectional study in Yueyang Hospital (affiliated with Shanghai University of Traditional Chinese Medicine) and Shanghai Skin Diseases Hospital (affiliated with Tongji University) during January to May 2021. The sample size was calculated based on the standard formula for a cross-sectional study:

*n*=[*μ_α_^2^* × *p*(1-*p*)]/*δ^2^*

Previous studies have indicated that the prevalence of psoriasis patients with MetS was 30% in China^24^ . In this study, we set *α*=0.05, *p*=30%, and *δ*=10% of *p*; the sample size calculation demonstrated that at least 897 psoriasis patients should be recruited. We then employed a cluster survey method to recruit all psoriasis patients during the study period with informed consent in Yueyang Hospital and Shanghai Skin Diseases Hospital. The investigators informed each psoriasis patients the purpose and content of the study before questionnaire interviews. This study was performed in accordance with the guidelines of the STROBE statement for observational studies and had been approved by the Ethics Review Committee of Yueyang Hospital, Shanghai University of Traditional Chinese Medicine (Approval number: 2019–028). In this study, we included 1014 psoriasis patients in the final analysis.

### Diagnosis, inclusion and exclusion criteria for psoriasis

In this study, the diagnosis of psoriasis was confirmed by Chinese clinical dermatology, with skin damage manifested as localized or systemic. The clinical symptoms of psoriasis are mainly red inflammatory papules, maculopapular rash, patches of varying sizes, which are covered with multiple layers of silvery-white scales^25^. After scraping the scale, there is a layer of bright film and punctate bleeding under the film. In this study, the inclusion criteria of psoriasis were patients who met the clinical criteria for psoriasis and were aged >18 years for both genders; psoriasis patients with neurological or psychiatric abnormalities were excluded.

### Data collection

Data were collected both by an electronic questionnaire and the clinical physical examination records during January to May 2021. Psoriasis patients were encouraged to complete a face-to-face questionnaire investigation, the main contents were as follows: 1) Basic information – gender, age, education level, personal monthly income etc.; 2) Life habits – tobacco smoking status (current/former smoker, age for tobacco smoking initiation, smoking duration, tobacco smoking per day), alcohol consumption, and tea drinking etc.; 3) Medical history of non-communicable diseases (NCD) – type 2 diabetes mellitus (T2DM), hypertension, hyperlipidemia, psoriasis history, psoriasis recurrence and family history; and 4) Lab examination – blood biochemical indicators. Physical examination including the height, weight, waist circumference, and other body measurement information of patients were extracted directly from their medical records in hospital. This study presents a homogenization of the management in the blood biochemical indicators between the two hospitals.

### Definition and diagnostic criteria for MetS

We followed the guidelines for the prevention and treatment of type 2 diabetes in China (2020 edition, and recently revised by the Chinese Diabetes Society) to evaluate the presence of MetS, and this proved to be a major contributing factor to this association^26^. Metabolic syndrome was defined for those individuals with ≥3 types of abnormalities which included hypertension, T2DM, increased waist circumference, hypertriglyceridemia, and reduced HDL. T2DM was defined as patients with FBG (Fasting Blood Glucose) ≥6.1 mmol/L or 2-h post prandial blood glucose ≥7.8 mmol/L in fasting. Hypertension was defined as patients with blood pressure ≥130/85 mmHg. Hypertriglyceridemia was verified if triglycerides ≥1.7 mmol/L, reduced HDL was defined as HDL cholesterol <1.04 mmol/L. The increased waist circumference was defined as waist circumference ≥90 cm in men and ≥85 cm in women.

### Determination of smoking status

We defined smokers as those who smoked at least one cigarette per day for over 6 months in their lifetime. We defined current smokers as those smokers who were still smoking at the time of the investigation, and former smokers as those smokers who had abstained from smoking for over 3 months at the time of the investigation. The smoking intensity was defined as the number of cigarettes smoked per day, and was categorized into: 1–9 cigarettes/day, 10–19 cigarettes/ day, and ≥20 cigarettes/day. Smoking duration was calculated as the age at investigation minus the age at which a smoker first started regular cigarette smoking; and smoking duration was divided into five groups: <10, 10–19, 20–29, 30–39, and ≥40 years. Age was stratified into: <30, 30–39, 40–49, 50–59, and ≥60 years.

### Classification of BMI, education status and personal monthly income

BMI (kg/m^2^)of psoriasis patients was categorized as follows: underweight (<18.50), normal weight (18.50–24.99), overweight (24–27.99), and obese (≥28). Education level was classified as junior high school or below, senior high school, and college degree or above. Personal monthly income was categorized into: <3000, 3000–5000, 5001–10000 and >10000 RMB (1000 Chinese Renminbi about US$160). The disease duration of psoriasis patients was classified as: <10, 10–19, 20–29, and ≥30 years.

### Statistical analysis

SPSS 24.0 (IBM, Armonk, NY, USA) and Prism 8.0 software (GraphPad Software, San Diego, CA, USA) were applied for statistical analysis. Quantitative variables with normal distribution were expressed as mean ± standard deviation (SD), and the t-test was used to test for significance between different groups. Quantitative variables with skewed distribution were expressed as median and interquartile range (IQR), and the non-parametric rank-sum test was applied to examine differences between groups. Qualitative variables were expressed as frequency counts and percentage (%), chi-squared test was used to determine statistical differences between groups.

Logistic regression was applied to calculate the odds ratios (OR) and 95% confidence interval (95% CI) to explore the association between tobacco smoking and MetS among psoriasis patients, with the adjustment of potential confounders identified by the directed acyclic graphs (DAGs). A p<0.05 (two-tailed) was considered statistically significant.

## RESULTS

In this cross-sectional study, we finally included 1014 psoriasis patients and grouped them into 261 psoriasis patients with MetS (25.74%) and 753 psoriasis patients without MetS (74.26%). Table 1 shows that the psoriasis patients were predominantly male (65.58%), with an average age of 45.98 years (IQR: 34.00–57.00). In all, 43.29% of psoriasis patients had an education level of college and above. The proportion of overweight or obese psoriasis patients was 52.77%. In addition, over half of psoriasis patients had a disease duration <10 years (54.24%); 22.29% of psoriasis patients had a family history of psoriasis diseases, and the proportion of alcohol drinking and tea drinking among psoriasis patients was 13.02% and 23.77%, respectively (Table 1).

**Table 1:** Demographic features of psoriasis patients with and without MetS ^a^, Shanghai, 2021 (N=1014)

*Characteristics*	*Total psoriasis patients n (%)*	*Psoriasis patients*		*p*
		*With MetS n (%)*	*Without MetS n (%)*	
**Total**, n	1014	261	753	
**Gender**				0.00
Male	665 (65.58)	195 (29.32)	470 (70.68)	
Female	349 (34.42)	66 (18.91)	283 (81.09)	
**Age** (years)				0.00
<30	147 (14.50)	2 (1.36)	145 (98.64)	
30–39	239 (23.57)	20 (8.37)	219 (91.63)	
40–49	180 (17.75)	65 (36.11)	115 (63.89)	
50–59	234 (23.08)	84 (35.90)	150 (64.10)	
≥60	214 (21.10)	90 (42.06)	124 (57.94)	
**Education level**				0.00
Junior high school or below	257 (25.35)	64 (24.90)	193 (75.10)	
Senior high school	318 (31.36)	115 (36.16)	203 (63.84)	
College degree or above	439 (43.29)	82 (18.68)	357 (81.32)	
**Personal monthly income** (RMB)				0.00
<3000	144 (14.20)	17 (11.81)	127 (88.19)	
3000–5000	289 (28.50)	72 (24.91)	217 (75.09)	
5001–10000	434 (42.80)	140 (32.26)	294 (67.74)	
>10000	147 (14.50)	32 (21.77)	115 (78.23)	
**BMI** (kg/m^2^)				0.00
<18.50	48 (4.73)	1 (2.08)	47 (97.92)	
18.50–23.99	431 (42.50)	73 (16.94)	358 (83.06)	
24.00–27.99	400 (39.45)	125 (31.25)	275 (68.75)	
≥28	135 (13.32)	62 (45.93)	73 (54.07)	
**Duration of psoriasis** (years)				0.01
<10	550 (54.24)	120 (21.82)	430 (78.18)	
10–19	232 (22.88)	75 (32.33)	157 (67.67)	
20–29	145 (14.30)	40 (27.59)	105 (72.41)	
≥30	87 (8.58)	26 (29.89)	61 (70.11)	
**Family history of psoriasis**				0.63
Yes	226 (22.29)	61 (26.99)	165 (73.01)	
No	788 (77.71)	200 (25.38)	588 (74.62)	
**Alcohol status**				0.01
Yes	132 (13.02)	46 (34.85)	86 (65.15)	
No	882 (86.98)	215 (24.38)	667 (75.62)	
**Tea status**				0.00
Yes	241 (23.77)	112 (46.47)	129 (53.53)	
No	773 (76.23)	149 (19.28)	624 (80.72)	
**Smoking**				
Yes	323 (31.85)	150 (46.44)	173 (53.56)	0.00
No	691 (68.15)	111 (16.06)	580 (83.94)	

a MetS: metabolic syndrome.

BMI: body mass index. RMB: 1000 Chinese Renminbi about US$160.

### Prevalence of MetS in psoriasis patients

Among 1014 psoriasis patients, 261 met the criteria of MetS, and the prevalence of MetS was 25.74% (29.32% in males and 18.91% in females). Psoriasis patients with education level of college and above had a lower prevalence of MetS, and the prevalence of MetS was lower among patients with monthly income <3000 RMB. Moreover, the prevalence of MetS increased with age, BMI and psoriasis duration, and psoriasis patients with tea drinking and/or alcohol drinking habits also had a higher prevalence of MetS (Table 1).

Table 2 shows that 74.56% of psoriasis patients had increased waist circumference, 31.85% had hypertension, 28.11% with triglyceride levels ≥1.7 mmol/L, 20.41% had Type 2 diabetes, and 5.03% with HDL <1.04 mmol/L. The proportion of increased waist circumference, hypertension, dyslipidemia and T2DM was higher among psoriasis patients with MetS than those without (Table 2).

**Table 2:** Factors associated with the metabolic syndrome Shanghai, 2021 (N=1014)

*Factors ^a^*	*Total psoriasis patients n (%)*	*Psoriasis patients*		*p*
		*With MetS n (%)*	*Without MetS n (%)*	
**Total**, n	1014	261	753	
Waist circumference (≥90 cm in men; ≥85 cm in women)	756 (74.56)	247 (94.64)	509 (67.60)	0.00
Triglyceride levels (≥1.7 mmol/L)	285 (28.11)	208 (79.69)	77 (10.23)	0.00
Low HDL^b^ (<1.04 mmol/L)	52 (5.03)	31 (11.87)	21 (2.79)	0.00
Hypertension (≥130/85 mmHg)	323 (31.85)	214 (81.99)	109 (14.48)	0.00
Type 2 diabetes FBG^c^ ≥6.1 mmol/L (or 2-h post prandial blood glucose ≥7.8 mmol/L in fasting)	207 (20.41)	158 (60.54)	49 (6.51)	0.00

a MetS: metabolic syndrome;

b HDL: high-density lipoprotein.

c FBG: fasting blood glucose.

### Association of smoking and MetS among psoriasis patients

In all, 323 of the 1014 psoriasis patients were smokers, and the prevalence of smoking was 31.85%. Male psoriasis patients had significantly higher tobacco smoking prevalence than female patients; with increasing age and BMI, the prevalence of tobacco smoking among psoriasis patients increased dramatically (p<0.01) (Table 3).

**Table 3:** The demographic feature of smoking psoriasis patients and non-smoking psoriasis patients, Shanghai, 2021 (N=1014)

*Characteristics*	*Smoking (n=323) n (%)*	*No smoking (n=691) n (%)*	*p*
**Gender**			0.00
Male	301 (45.26)	364 (54.74)	
Female	22 (6.30)	327 (93.70)	
**Age** (years)			0.00
<30	28 (19.05)	119 (80.95)	
30–39	66 (27.62)	173 (72.38)	
40–49	58 (32.22)	122 (67.78)	
50–59	91 (38.89)	143 (61.11)	
≥60	80 (37.38)	134 (62.62)	
**BMI**^a^ (kg/m^2^)			0.00
<18.50	7 (14.58)	41 (85.42)	
18.50–23.99	113 (26.22)	318 (73.78)	
24.00–27.99	147 (36.75)	253 (63.25)	
≥28	56 (41.48)	79 (58.52)	
**Education level**			0.09
Junior high school or below	95 (36.96)	162 (63.04)	
Senior high school	101 (31.76)	217 (68.24)	
College degree or above	127 (28.93)	312 (71.07)	
**Personal monthly income** (RMB)			0.40
<3000	37 (25.69)	107 (74.31)	
3000–5000	95 (32.87)	194 (67.13)	
5001–10000	142 (32.72)	292 (67.28)	
>10000	49 (33.33)	98 (66.67)	

a BMI: body mass index.

RMB: 1000 Chinese Renminbi about US$160.

Tables 4 shows that psoriasis patients with smoking had a higher prevalence of MetS than those without tobacco smoking (OR=2.26; 95% CI: 1.68–3.02). Multivariate logistic regression indicated that psoriasis patients with tobacco smoking had 1.78 times (95% CI: 1.21–2.60) the probability to have MetS than those without smoking, after adjusting for gender, age, education level, monthly income, BMI, alcohol drinking, psoriasis duration, and tea drinking habit (Table 4).

**Table 4:** The association between smoking status and psoriasis patients with MetS ^a^ (N=261) compared with psoriasis alone (N=753) by logistic regression analysis, Shanghai, 2021

*Variables*	*Univariate model*		*Multivariate model ^c^*	
	*OR*	*95% CI*	*AOR*	*95% CI*
**Smoking status**, yes vs no	2.26	1.68–3.02	1.78	1.21–2.60
**Gender**, male vs female	1.78	1.30–2.44	1.14	0.74–1.74
**Age** (years)				
<30	1		1	
30–39	6.62	1.52–28.76	4.37	0.98–19.54
40–49	40.98	9.82–170.93	26.84	6.23–115.67
50–59	40.60	9.81–168.10	23.88	5.53–103.06
≥60	52.62	12.7–218.04	33.34	7.68–144.76
**Education level**				
Junior high school or below	1.44	1.00–2.09	1.22	0.72–2.07
Senior high school	2.47	1.77–3.44	1.59	1.03–2.47
College degree or above	1		1	
**Personal monthly income** (RMB)				
<3000	1		1	
3000–5000	2.48	1.40–4.39	1.87	0.97–3.61
5001–10000	3.56	2.06–6.13	3.21	1.63–6.34
>10000	2.08	1.10–3.94	2.48	1.08–5.71
**BMI**^b^ (kg/m^2^)				
<18.50	1		1	
18.50–23.99	9.58	1.30–70.58	6.08	0.79–47.08
24.00–27.99	21.36	2.92–156.59	9.81	1.27–75.85
≥28	39.92	5.35–297.75	26.62	3.37–210.55
**Duration of psoriasis** (years)				
<10	1		1	
10–19	1.71	1.22–2.41	1.47	0.97–2.20
20–29	1.37	0.90–2.07	0.88	0.54–1.41
≥30	1.53	0.93–2.52	0.84	0.47–1.47
**Alcohol status**, yes vs no	1.66	1.12–2.45	1.34	0.81–2.20
**Tea status**, yes vs no	3.64	2.67–4.96	2.47	1.69–3.61

a MetS: metabolic syndrome;

b BMI: body mass index. RMB: 1000 Chinese Renminbi about US$160.

c Adjusted for gender, age, education, monthly income, BMI, alcohol drinking, psoriasis duration and tea drinking habit.

AOR: adjusted odds ratio.

### Tobacco smoking condition among psoriasis patients with and without MetS

**Figure 1:**
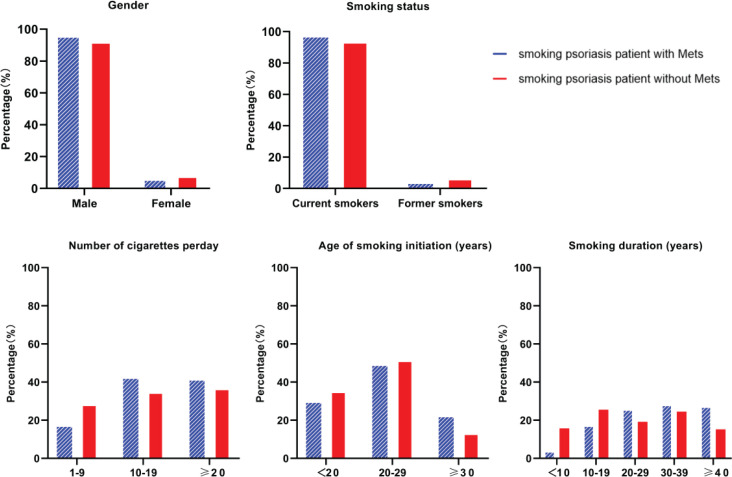
Smoking status and demographic characteristics of psoriasis patients with or without MetS

The 323 psoriasis patients with tobacco smoking included 301 males (93.19%), with an average age of 49.13 years (SD: 13.46). Among the 323 psoriasis patients with tobacco smoking, 93.19% were current smokers, the median number of daily consumed cigarettes was 12 (IQR: 10–20), 33.13% initiated tobacco smoking at age <20 years, and approximately 90% had a smoking duration >10 years. Figure 1 shows that smoking psoriasis patients with MetS had a higher proportion of males, were more likely to be a current smoker, consumed more cigarettes per day, with longer smoking duration, but with older age of smoking initiation (Figure 1).

## DISCUSSION

Studies have indicated that tobacco smoking was an independent risk factor both for the development of psoriasis and metabolic syndrome diseases^22,23^. Nevertheless, the effect of tobacco smoking on the co-morbidities prevalence of psoriasis and metabolic syndrome has not been adequately studied. In this study, we found that 25.74% of psoriasis patients had metabolic syndrome; this finding is in line with a large cross-sectional study conducted in Spain (28.3%)^21^, but the prevalence of MetS in psoriasis was lower than other studies in China (37.56% and 38.1%, respectively)^27,28^. These inconsistencies may be due to the age structure difference of participants, and the smaller sample size in these studies. In addition, the prevalence of metabolic syndrome was higher among patients with psoriasis compared to the general population in Shanghai (20.2% in men and 18.7% in women), which indicates that psoriasis is closely related to MetS.

It is well known that MetS is strongly associated with age, and is also treated as a marker for rapid aging^29,30^. Findings in this study demonstrate that psoriasis patients aged ≥40 years have significantly higher prevalence of MetS, which is consistent with previous published results.

Smoking was confirmed to be an important environmental risk factor that was positively associated with an increased prevalence of metabolic syndrome in psoriasis patients. We divided psoriasis patients into psoriasis with and without MetS. We found that the prevalence of smoking was 31.85% in psoriasis patients and 36.84% in psoriasis patients combined with metabolic syndrome; the psoriasis patients with MetS had a higher prevalence of tobacco smoking than those without MetS, which is in line with previous studies. Moreover, our previous study found that the smoking prevalence among the general population in the same area was only 19.78%^31^, which suggested that smoking might be a risk factor both for psoriasis and metabolic syndrome. It has been reported that both psoriasis and metabolic syndrome are genetically related diseases, but we did not find a statistically significant difference in the family history between psoriasis patients with MetS and without MetS, which might be due to the small population size in this study.

We evaluated if tobacco smoking was responsible for the increased risk of metabolic syndrome among psoriasis patients. We found a positive association between the prevalence of metabolic syndrome and smoking duration among psoriasis patients. Figure 1 shows that the percentage of psoriasis patients with metabolic syndrome increased with increasing tobacco smoking duration; this might be due to the fact that long-term cigarette smoking induces a recurrent inflammatory response and passive activation of the immune system, which in turn further impairs blood circulation and promotes an increase in coagulation and endothelial cells, and results in higher prevalence of MetS in smokers.

Despite many conflicting findings, there is a global consensus that smoking has negative health effects and increases the severity of psoriasis. Tobacco smoking is a recognized risk factor for many chronic diseases such as cardiovascular diseases, respiratory diseases, autoimmune diseases, and allergies (such as dermatitis, eczema, urticaria). Complex components of cigarette smoke play roles in regulating immunity by exacerbating pathogenic immune responses or attenuating defensive immunity by affecting adaptive immune cells (Th1, Th2, Th17, CD4+, CD8+, B cells and memory T/B lymphocytes) and innate immune cells (dendritic cells, macrophages and NK cells). Smoking is a well-known environmental trigger for psoriasis and associated with the progression of metabolic syndrome. Many studies have reported a strong association between smoking and the development of the metabolic syndrome. However, the exact mechanism is still unknown^32,33^. Potential mechanisms underlying the relationship between tobacco smoking and psoriasis patients with MetS includes the stimulation of keratinocyte differentiation, reduction of antioxidant levels, and induction of T-cell proliferation^34,35^.

Obesity has become one of the major health problems which is strongly associated with the development of diabetes, hypertension, and hyperlipidemia. In this study, psoriasis patients with MetS had a higher percentage of BMI ≥24 than those without MetS, and BMI was positively correlated with the higher prevalence of MetS among psoriasis patients. The pathogenesis of obesity and MetS maybe that adipose tissue contributes to the development of MetS by adipokines which promote inflammation, affect glucose metabolism, and promote vascular endothelial proliferation. Lipocalin production by adipocytes is negatively correlated with BMI, and low lipocalin levels promote vascular damage and chronic inflammation, which may underlie the pathology leading to the development of obesity and psoriasis co-morbidities and therefore play a key role in the pathogenesis of the MetS. Researchers suggest that weight loss may improve the symptoms of psoriasis by lowering the circulation levels of inflammatory cytokines caused by obesity^36^.

Interestingly, this study showed that tea consumption was associated with MetS among psoriasis patients, and the prevalence of MetS was higher in psoriasis patients with tea drinking than those without. The positive association between tea consumption and higher prevalence of MetS in psoriasis patients maybe a ‘concomitant phenomenon’, as a high proportion of the tea drinkers were male psoriasis patients, aged ≥50 years, were overweight or obese, and had a higher MetS prevalence in this study.

### Limitations

There are some limitations in this study. First, we recruited psoriasis patients only in two hospitals in Shanghai, so the findings have limited representation of the total psoriasis patients. Second, indicators for psoriasis severity analysis was not included in this study, so we missed the chance to evaluate the dose-response relationship between smoking intensity and the severity of psoriasis; the incorporation of indicators for psoriasis severity and life quality including Psoriasis Area and Severity Index, Body Surface Area, Physician’s Global Assessment, Dermatology Life Quality Index, Professional Quality of Life Scale and Visual Analogue Scale should be considered in future study. Third, information such as tobacco consumption among former smokers, smoking status, alcohol drinking and tea drinking were collected through face-to-face interviews, which might have led to recall bias. All of these limitations might restrict the interpretations of the clinical findings to some degree. A better designed cohort or case-control study to explore the potential mechanism of smoking behavior in psoriasis patients with MetS may be useful for future research directions and intervention strategies.

## CONCLUSIONS

This study indicates that the prevalence of tobacco smoking and Mets among psoriasis patients is high in Shanghai, and tobacco smoking is positively associated with the metabolic syndrome among psoriasis patients. Clinicians should recommend that psoriasis patients abstain from tobacco smoking and provide tobacco cessation assistance regularly.

## Data Availability

The data supporting this research are available from the authors on reasonable request.
